# Arginase-1 Deficiency Regulates Arginine Concentrations and NOS2-Mediated NO Production during Endotoxemia

**DOI:** 10.1371/journal.pone.0086135

**Published:** 2014-01-21

**Authors:** Karolina A. P. Wijnands, Marten A. Hoeksema, Dennis M. Meesters, Nynke M. S. van den Akker, Daniel G. M. Molin, Jacob J. Briedé, Mitrajit Ghosh, S. Eleonore Köhler, Marc A. M. J. van Zandvoort, Menno P. J. de Winther, Wim A. Buurman, Wouter H. Lamers, Martijn Poeze

**Affiliations:** 1 Department of Surgery, Maastricht University Medical Centre, Maastricht, the Netherlands; 2 NUTRIM School for Nutrition, Toxicology and Metabolism, Maastricht University Medical Centre, Maastricht, the Netherlands; 3 Department of Medical Biochemistry, Academic Medical Center, University of Amsterdam, Amsterdam, the Netherlands; 4 Department of Cardiology, Maastricht University Medical Centre, Maastricht, the Netherlands; 5 CARIM Cardiovascular Research Institute of Maastricht, Maastricht University Medical Centre, Maastricht, the Netherlands; 6 Department of Physiology, Maastricht University Medical Centre, Maastricht, the Netherlands; 7 Department of Toxicogenomics, Maastricht University Medical Centre, Maastricht, the Netherlands; 8 GROW School for Oncology and Developmental Biology, Maastricht University Medical Centre, Maastricht, the Netherlands; 9 Department of Genetics & cell Biology, Section Molecular Cell Biology, Maastricht University Medical Centre, Maastricht, the Netherlands; 10 Department of Anatomy & Embryology, Maastricht University Medical Centre, Maastricht, the Netherlands; Indian Institute of Science, India

## Abstract

**Rationale and objective:**

Arginase-1 is an important component of the intricate mechanism regulating arginine availability during immune responses and nitric oxide synthase (NOS) activity. In this study *Arg1^fl/fl^/Tie2-Cre^tg/−^* mice were developed to investigate the effect of arginase-1 related arginine depletion on NOS2- and NOS3-dependent NO production and jejunal microcirculation under resting and endotoxemic conditions, in mice lacking arginase-1 in endothelial and hematopoietic cells.

**Methods and Results:**

Arginase-1-deficient mice as compared with control mice exhibited higher plasma arginine concentration concomitant with enhanced NO production in endothelial cells and jejunal tissue during endotoxemia. In parallel, impaired jejunal microcirculation was observed in endotoxemic conditions. Cultured bone-marrow-derived macrophages of arginase-1 deficient animals also presented a higher inflammatory response to endotoxin than control littermates. Since NOS2 competes with arginase for their common substrate arginine during endotoxemia, *Nos2* deficient mice were also studied under endotoxemic conditions. As *Nos2^−/−^* macrophages showed an impaired inflammatory response to endotoxin compared to wild-type macrophages, NOS2 is potentially involved. A strongly reduced NO production *in Arg1^fl/fl^/Tie2-Cre^tg/−^* mice following infusion of the NOS2 inhibitor 1400W further implicated NOS2 in the enhanced capacity to produce NO production *Arg1^fl/fl^/Tie2-Cre^tg/−^* mice.

**Conclusions:**

Reduced arginase-1 activity in *Arg1^fl/fl^/Tie2-Cre^tg/−^* mice resulted in increased inflammatory response and NO production by NOS2, accompanied by a depressed microcirculatory flow during endotoxemia. Thus, arginase-1 deficiency facilitates a NOS2-mediated pro-inflammatory activity at the expense of NOS3-mediated endothelial relaxation.

## Introduction

Arginase plays an important role in the regulation of L-arginine availability for the nitric oxide (NO) production in the circulation [1]–[4] and the immune response [5]–[14]. Under normal conditions, sufficient L-arginine is available in endothelial cells to convert it to NO and L-citrulline by endothelial nitric oxide synthase (NOS3; eNOS). The enzyme arginase competes with the NOS enzymes for L-arginine as substrate and reciprocally modulates the NOS activity [15], [16]. There are two isoforms of arginase, of which arginase-1 mainly expressed in the liver and arginase-2, a mitochondrial enzymes is primarily expressed in non-hepatic tissue such as the kidney, small intestine [8] and endothelial cells [17]. Inflammatory conditions result in significantly decreased L-arginine concentrations for NOS3 because of a pathogen-induced upregulation of arginase-1 and NOS2 (iNOS) in macrophages [1], [10], [13], [18]–[20]. The arginase-mediated decreased in L-arginine concentrations and the endotoxin-induced downregulation of NOS3 further impair NOS3-derived NO bioavailability in the microvasculature [11], [21], [22], which results in endothelial dysfunction [23]–[27].

Reduction or complete ablation of arginase-1 activity is known to significantly enhance NOS-dependent NO production [2], [28], [29]. Therefore, modulations of arginase-1 activity may be an interesting therapeutic option to treat inflammatory conditions such as sepsis, which are characterized by an impaired NOS3-derived NO production [1], [2], [11]–[13], [22].

Here, we investigate the role of arginase-1 in regulating the L-arginine bioavailability for NO production in macrophages and endothelial cells during endotoxin-induced inflammation. We hypothesized that tissue-specific arginase-1 deletion will prevent the reduction of cellular arginine substrate and conserve NOS3-dependent NO production and, hence, jejunum perfusion. In addition to the effects on NOS3 activity, we also expected that NOS2-dependent NO production would increase if arginase-1 was deleted in inflammatory cells such as macrophages. To study the potential inter-relationship between arginase-1, NOS2 and NOS3 activity, mice deficient for arginase-1 in endothelial and hematopoietic cells were developed [30] and were exposed to a prolonged endotoxemia model as previously developed by our group [31].

## Materials and Methods

### Animals

To investigate the role of arginase-1, *Arg^fl/fl^/Tie2-Cre^tg/−^* mice were generated in our laboratory as described in detail elsewhere [30], [32]. In brief, *Arg1* exon 4 on chromosome 10 was surrounded by LoxP sites [32], as exon 4 is essential for the enzymatic activity of arginase1 [33] and deletion will result in a frame shift. The targeting vector (17.515 Kb) used consisted of *Arg1* exons and introns 2 and 3 (4.6 Kb) at the 5′end, a Neo-TK selection cassette flanked by frt sites [34], exon 4 (160 bp) flanked by loxP sites [35], introns 4–7, exons 5–8, and a small fragment downstream of the gene (in total 4.3 Kb) at the 3′end. The targeting construct was sequence-verified with respect to exons, splice junctions, and recombinase-recognition sites, digested with AscI (introduced by PCR for cloning purposes), and purified by electrophoresis and electroelution. The final targeting construct was electroporated into mouse ES cell line E14IB10 (129/Ola). Southern blotting and long-distance PCR followed by sequencing demonstrated the proper recombination with the *Arg1* allele on mouse chromosome 10. After a “deep split” to ensure the presence of recombined ES clones only, the Neo-TK cassette was removed by transient transfection with an FLPe recombinase expression vector (kindly provided by Dr. Francis Stewart, EMBL, Germany). Chimeric male mice were generated by injecting the *Arg1*-recombinant ES cells into C57Bl/6J blastocysts. These chimeric male mice were bred with female 129P2/OlaHsd mice (Harlan, The Netherlands). Mice were genotyped with primers Arg1-F and Arg1-R (**Table S1 in File S1**), yielding a 351 bp wild-type allele and a 384 bp floxed allele. The Cre-excised allele (298 bp) was detected by PCR with the primers Arg1-F2 (**Table S1 in File S1**) and Arg1-R1. To specifically delete the floxed *Arg1* allele (Arg1fl) in endothelial and hematopoetic cells, mice were crossed Tie2-Cre [36]. *Arg^fl/fl^* and *Tie2-Cre^tg/-^* mice were backcrossed more than 10 generations into the C57BL/6J background. Animals were genotyped for the presence of Tie2-Cre by PCR with primers Tie2-F and Tie2-R (**Table S1 in File S1**), yielding a 510 bp band for Cre-positive animals.

C57BL/6J *Arg1^fl/fl^* female mice were crossed with C57BL/6J *Arg^fl/fl^*/Tie2-Cre male mice resulting in *Arg1^fl/fl^/Tie2-Cre^−/−^* (littermates with no deletion of arginase-1, here described as control mice) and *Arg1^fl/fl^/Tie2-Cre^tg/−^* mice with depletion of arginase-1 in all hematopoietic cells.

Since murine erythrocytes do not express arginase-1 and to our knowledge expression in lymphocytes has not been described, this leaves myeloid and endothelial cells. We limit our discussion to macrophages and endothelial cells, which are the most relevant cells for vascular NO-production.

In total, 59 mice (27 control and 32 *Arg1^fl/fl^/Tie2-Cre^tg/−^*) weighing between 25–30 g were obtained from the Central Animal Facilities of the Maastricht University. Since arginase-1 and NOS2 compete for the same substrate, and, therefore, impair the arginine availability for NOS3, *Nos2^−/−^* mice [37] (n = 17), also backcrossed more than 10 generations into the C57BL/6J background, were obtained from the VIB Department for Molecular Biomedical Research, University Ghent. All mice were individually housed at room temperature, maintained on a 12 hour light-dark cycle, fed standard lab chow (Hope Farms, Woerden, the Netherlands) and water *ad libitum* until the experimental phase of the study.

The protocol was approved by the Committee on the Ethics of Animal Experiments of the Maastricht University Medical Center (Permit Number: 2010-172). All animal work has been conducted according to relevant national and international guidelines.

### Experimental setup

The experimental protocol and surgical procedures were described in detail before [31]. In brief, a jugular vein catheter (applied to 67 mice; 24 control mice, 29 *Arg1^fl/fl^/Tie2-Cre^tg/−^* and 14 *Nos2^−/−^* mice) provided access for a subsequent continuous infusion. Four days after cannulation, these mice were attached to a swivel system for an 18-hour continuous infusion (83μL/h, 1.5 mL in total) and were randomly allocated to receive lipopolysaccharide (LPS; E. Coli O55:B5, Sigma Aldrich, St. Louis, MO, 0.4µg/g bodyweight^−1^•h^−1^; n = 36) or sterile 0.9% saline (n = 31). At the end of the 18-hour infusion period, mice were anesthetized as described [31] for a second surgical procedure to measure jejunal microcirculation (n = 42, 7 mice per condition per mouse strain) and *in vivo* tissue NO levels (n = 25, 5 mice per condition per mouse strain) as described in detail before [31]. A brief description of the measurements of the jejunal microcirculation and the *in vivo* tissue NO production can be found in the **Supplementary Materials and Methods section in File S1**. The total number of villi analyzed did not differ between the control and *Arg1^fl/fl^/Tie2-Cre^tg/−^* mice under basal and endotoxemic conditions. To determine whether tissue-specific *Arg-1* deficiency during endotoxemia enhanced NOS3-derived NO production, the effect of NOS2 on the total NO production was suppressed by administering a selective NOS2 inhibitor 1400W [38] (20 mg/kg) intraperitoneally 4 hours prior to the measurements (*Arg1^fl/fl^/Tie2-Cre^tg/−^* + LPS + 1400W; n = 5).

To define the isolated effect of macrophage arginase-1 for the arginine-NO pathway, 12 mice, 6 controls (3 *Arg1^fl/fl^/Tie2-Cre^−/−^* and 3 *Nos2^+/−^* mice), 3 *Arg1^fl/fl^/Tie2-Cre^tg/−^* and 3 *Nos2^−/−^* mice were used to obtain bone marrow-derived macrophages (BMMs) for *in vitro* experiments. A detailed description of this experiment, analysis of the *in vitro* NO production with Griess reagent and cytokine measurements in BMM supernatants can be found in the **Supplementary Materials and Method section in File S1**.

At the end of the microcirculatory or *in vivo* NO measurements, mice were euthanized via a cardiac puncture to sample arterial blood. Jejunal tissue was harvested and either fixed in 4% formaldehyde and embedded in paraffin for immunohistochemistry or snap-frozen in liquid nitrogen, and stored at −80°C till further analysis. Quantitative PCR, Western-blot protein analysis, plasma and tissue amino-acid and polyamine analysis with fully automated liquid chromatography-mass spectrometry system (LC-MS) are described briefly in the online supplemental material and method section. See the online **Table S1 in File S1** for the primers used for genotyping and quantitative PCR.

To determine endothelial NO production in our model, carotid arteries were isolated and immediately mounted in a pressure chamber. *Ex vivo* NO production in endothelial cells was detected with Cu_2_Fl_2_ using 2-photon imaging as described in the online **Supplementary Materials and Method section in File S1** [39].

### Statistical analysis

Statistical analysis of the data was performed using SPSS 15.0 (SPSS, Chicago, IL). One-way analysis of variance (ANOVA) was performed followed by a post-hoc Bonferroni correction between groups to determine significant differences. Data are represented as means and standard errors of the mean (SEM). P–values below 0.05 were considered as statistically significant.

## Results

### Effects of LPS on plasma and tissue amino-acid concentrations in *Arg1^fl/fl^/Tie2-Cre^tg/−^* mice

**Figure 1:**
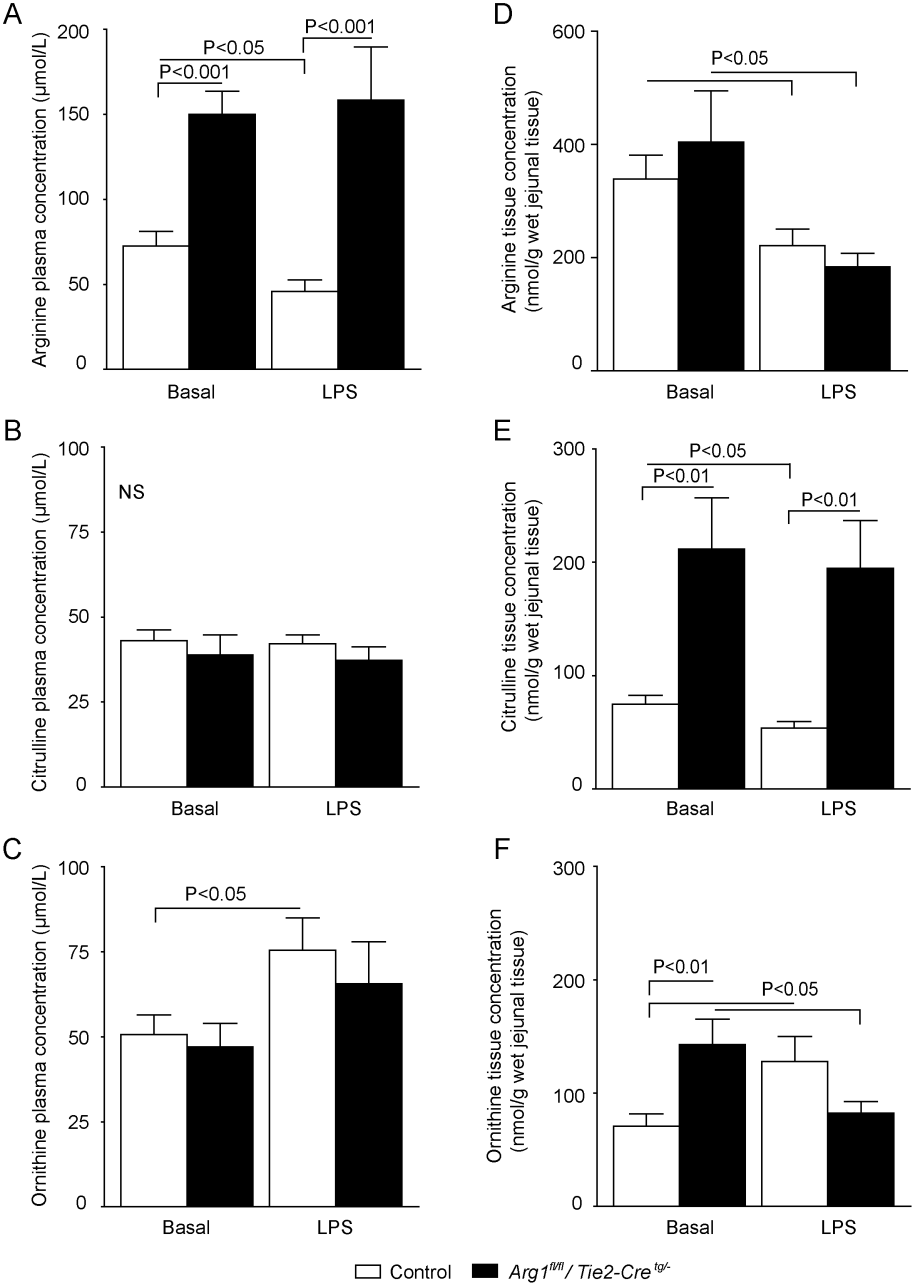
Plasma and tissue arginine, citrulline and ornithine concentrations in control and *Arg1^fl/fl^/Tie2-Cre^tg/−^* mice under basal and endotoxemic conditions. (A) *Arg1^fl/fl^/Tie2-Cre^tg/−^* mice had a ∼2-fold higher plasma arginine concentration than control mice (control and control + LPS), which was not affected by LPS infusion (*Arg1^fl/fl^/Tie2-Cre^tg/−^* + LPS). Plasma arginine concentration was significantly reduced in the control + LPS group compared to basal condition (P<0.05). (B) Plasma citrulline concentration was similar in control and *Arg1^fl/fl^/Tie2-Cre^tg/−^* mice under both basal and inflammatory conditions. (C) Compared to the basal condition, plasma ornithine concentration only significantly increased in the LPS-treated control group, but not the *Arg1^fl/fl^/Tie2-Cre^tg/−^* group. (D) Tissue arginine concentration declined ∼2-fold in LPS-treated groups compared to controls (P<0.05). (E) Tissue citrulline concentration was significantly higher in *Arg1^fl/fl^/Tie2-Cre^tg/−^* than control mice under basal and endotoxemic conditions (P<0.01). LPS infusion reduced citrulline concentration in control (P<0.05), but not in *Arg1^fl/fl^/Tie2-Cre^tg/−^* mice. (F) Under basal conditions, tissue ornithine concentration was higher in *Arg1^fl/fl^/Tie2-Cre^tg/−^* than control mice. After LPS infusion, ornithine concentration increased in control mice (P<0.05), but decreased in *Arg1^fl/fl^/Tie2-Cre^tg/−^* mice.

The tissue-specific arginase-1 knock-out mice (*Arg1^fl/fl^/Tie2-Cre^tg/−^*), with a deficiency of arginase-1 in macrophages and endothelial cells, exhibited significantly higher plasma concentrations of arginine both under basal conditions (saline infusion; P<0.001, n = 7; **Figure 1A** **,** **Table 1**) and after 18-hour continuous LPS infusion (P<0.001, n = 7) than control mice. While plasma arginine concentrations in control mice were significantly decreased after LPS infusion compared with basal conditions (**Figure 1A** **,** **Table 1**), the plasma arginine concentrations in *Arg1^fl/fl^/Tie2-Cre^tg/−^* mice (*Arg1^fl/fl^/Tie2-Cre^tg/−^* + LPS) were similar compared to basal conditions.

**Table 1:** Plasma arginine, citrulline and ornithine amino-acid concentrations in control and *Arg1^fl/fl^/Tie2-Cre^tg/−^* mice under basal and endotoxemic conditions.

	**Basal**		**LPS**	
	**Control**	***Arg1^fl/fl^/Tie2-Cre^tg/−^***	**Control**	***Arg1^fl/fl^/Tie2-Cre^tg/−^***
**Arginine**	72.6 ± 8.7^ $^	150.1 ± 13.5	45.9 ± 6.7^*^^#^	158.5 ± 31.3
**Citrulline**	43.1 ± 3.2	38.9 ± 5.9	42.2 ± 2.6	37.3 ± 4.0
**Ornithine**	50.7 ± 5.8	47.1 ± 6.9	75.5 ± 9.5^*^	65.7 ± 12.3

Plasma concentrations are displayed in µmol/L. Values are represented as mean ± SEM. Significance: *p<0.05 vs. control during basal conditions; $p<0.001 vs. *Arg1^fl/fl^/Tie2-Cre^tg/−^*during basal conditions; ^#^p<0.001 vs. *Arg1^fl/fl^/Tie2-Cre^tg/−^+ LPS*.

Next, we determined citrulline concentrations as citrulline is both a product of L-arginine metabolism by NOS and a substrate for L-arginine *de novo* synthesis. Interestingly, plasma citrulline concentrations were comparable between *Arg1^fl/fl^/Tie2-Cre^tg/−^* and control mice under both basal (∼40µmol/L, P = 0.5, n = 7) and endotoxemic conditions (P = 0.8, n = 7; **Figure 1B** **,** **Table 1**), despite the observed differences in plasma arginine concentrations.

Also, plasma ornithine concentrations, produced by arginase from L-arginine catabolism, were not different between *Arg1^fl/fl^/Tie2-Cre^tg/−^* mice and control mice under physiological conditions (P = 0.7, n = 7, **Figure 1C** **,** **Table 1**). LPS administration in *Arg1^fl/fl^/Tie2-Cre^tg/−^* mice was not associated with increased plasma ornithine concentrations compared with basal conditions (P = 0.2, n = 7). In contrast, a significantly higher (∼1.5 fold) ornithine plasma concentration was observed in control mice treated with LPS versus basal conditions (P<0.05, n = 7). Both sets of data indicate that arginase-1 is the dominant arginase isoform that converts arginine into ornithine under inflammatory conditions.

### Unaltered jejunal arginine concentrations in *Arg1^fl/fl^/Tie2-Cre^tg/−^* mice

**Figure 2:**
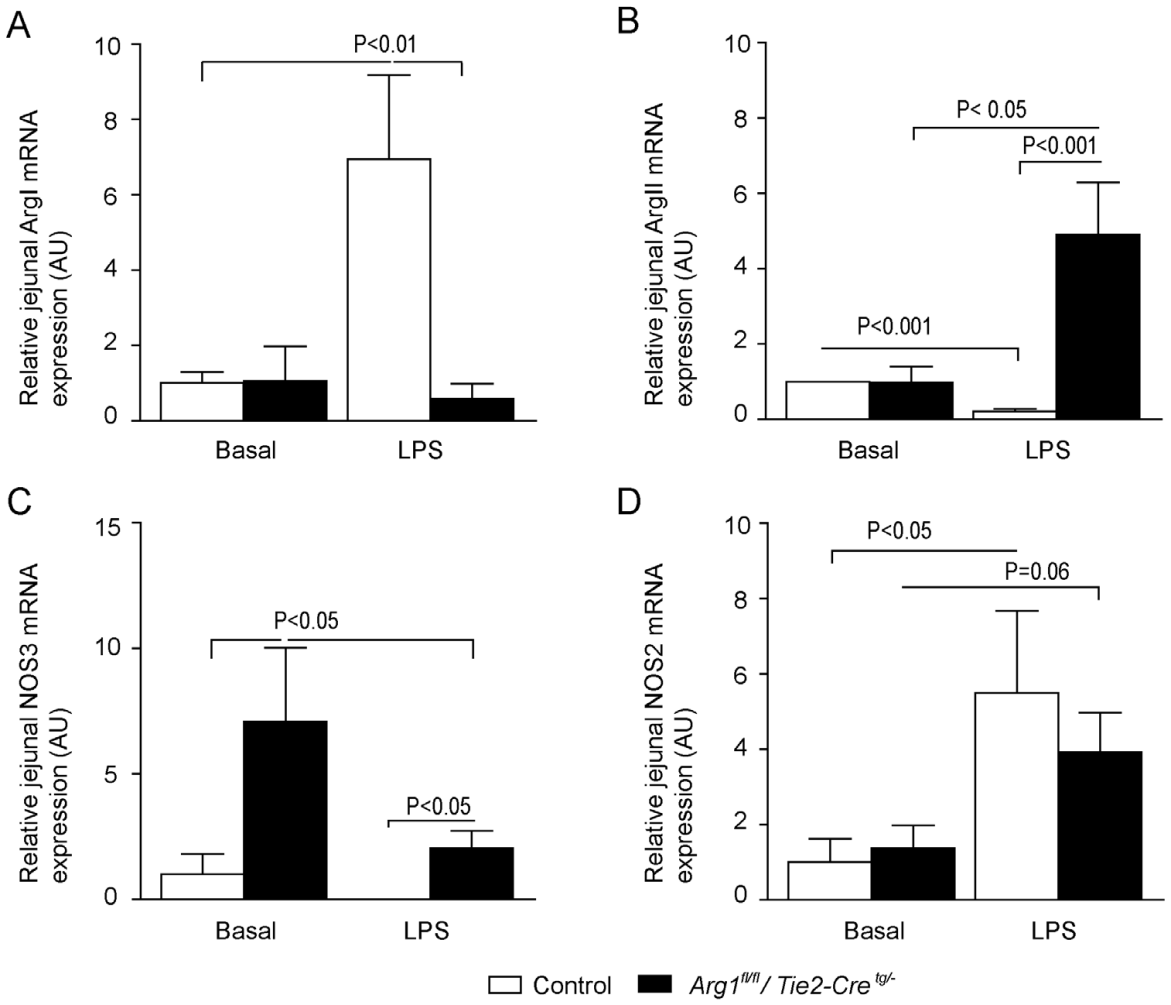
Relative mRNA expression in control and *Arg1^fl/fl^/Tie2-Cre^tg/−^* mice under physiological and endotoxemic conditions. (A) Relative *Arg1* mRNA expression was significantly higher in the control + LPS group than in basal and *Arg1^fl/fl^/Tie2-Cre^tg/−^* + LPS groups. As expected, *Arg1* expression was low in *Arg1^fl/fl^/Tie2-Cre^tg/−^* mice. (B) Interestingly, the relative *Arg2* mRNA expression was significantly higher in the *Arg1^fl/fl^/Tie2-Cre^tg/−^* mice after LPS infusion compared to control mice during endotoxemic conditions. (C) LPS infusion resulted in increased *Nos2* expression in both mouse strains compared to basal conditions. (D) *Nos3* mRNA expression was significantly higher in the *Arg1^fl/fl^/Tie2-Cre^tg/−^* mice compared to control animals under basal and LPS-infused conditions (p<0.05). The relative expression of the two household genes *Actb* and *Ppia* were used to calculate the geometric mean, which served as a normalization factor.

In contrast to plasma concentrations, the arginine concentration in jejunal tissue under basal conditions was similar between *Arg1^fl/fl^/Tie2-Cre^tg/−^* and control mice (**Figure 1D**). During LPS infusion, jejunal arginine concentrations in both mouse strains were significantly decreased (both P<0.05, n = 7), probably indicating an enhanced arginine catabolism during endotoxemia and a reduced arginine uptake [11], [12]. The increase in jejunal *Arg-1* mRNA abundance after LPS treatment in control mice probably explains the reduced arginine levels during endotoxemia (**Figure 2A**). As for *Arg1^fl/fl^/Tie2-Cre^tg/−^* mice, the reduced jejunal arginine levels may result from the increased *Arg-2* mRNA abundance after LPS treatment (**Figure 2B**), which may be upregulated as a compensatory mechanism in absence of arginase-1. In endotoxemic *Arg1^fl/fl^/Tie2-Cre^tg/−^* mice, an increased jejunal citrulline concentration was observed under both basal and endotoxemic conditions, indicating increased NOS activity (both P<0.01, n = 7; **Figure 1E**). Only in control mice did LPS infusion induce a 30% decrease of jejunal citrulline concentration (P<0.05, n =  7).

Basal ornithine concentration in jejunal tissue was ∼2-fold higher in *Arg1^fl/fl^/Tie2-Cre^tg/−^* than control mice (P<0.01, n = 7; **Figure 1F**). After LPS administration in control mice, a significant increase of the intracellular ornithine concentrations was observed compared with basal conditions. This increase in ornithine concentration is in line with the observed increase in plasma ornithine concentrations of the LPS-treated control mice (P<0.05, n = 7). In contrast, LPS administration in *Arg1^fl/fl^/Tie2-Cre^tg/−^* mice resulted in a significantly lower tissue ornithine concentration (P<0.05, n = 7), paralleled by a higher concentration of the polyamine putrescine (**Figure S1 in File S1**).

### LPS treatment increases jejunal NOS2 and NOS3 activity and NO concentrations in *Arg1^fl/fl^/Tie2-Cre^tg/−^* mice

**Figure 3:**
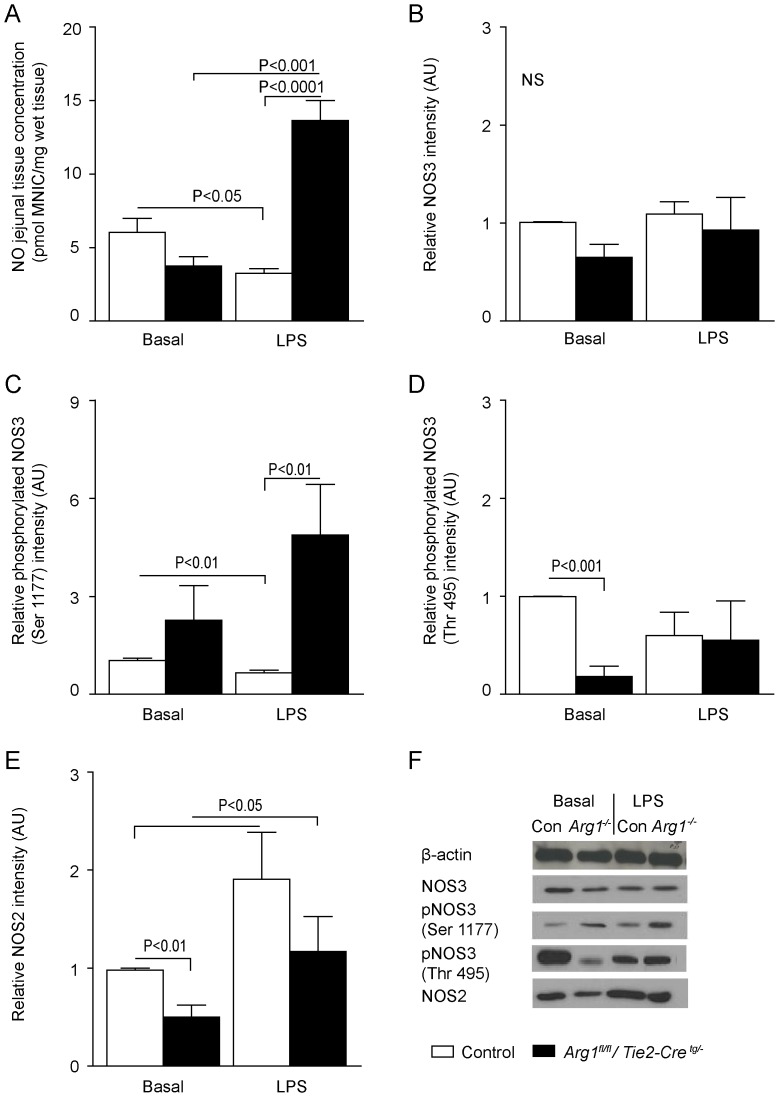
Jejunal NO production and NOS3 activity in control and *Arg1^fl/fl^/Tie2-Cre^tg/−^* mice. (A) In the jejunum, LPS treatment decreased *in vivo* NO production (measured as pmol mono-nitrosyl-iron complexes (MNIC)/mg wet tissue weight) in control mice to ∼50% (Basal vs. LPS, P<0.05; n = 5), whereas it increased NO production ∼3-fold in *Arg1^fl/fl^/Tie2-Cre^tg/−^* (Basal vs. LPS; P<0.001, n = 5). (B) Jejunal NOS3 protein concentration did not differ between basal and LPS-treated control or *Arg1^fl/fl^/Tie2-Cre^tg/−^* mice. (C) The degree of phosphorylation of NOS3 protein on Ser1177 decreased upon LPS treatment in control mice (P<0.01), but tended to increase in *Arg1^fl/fl^/Tie2-Cre^tg/−^* mice, so that the degree of jejunal NOS phosphorylation in LPS-treated *Arg1^fl/fl^/Tie2-Cre^tg/−^* mice was ∼5-fold higher than in control mice (p<0.01). (D) The degree of phosphorylation of NOS3 protein on Thr495 was significantly decreased during basal conditions in *Arg1^fl/fl^/Tie2-Cre^tg/−^* mice. (E) LPS treatment increased NOS2 protein concentration ∼2-fold in control and *Arg1^fl/fl^/Tie2-Cre^tg/−^* mice. NOS2 protein content was normalized with β-actin. (F) Representative Western blots of β-actin, NOS3, phosphorylated NOS3 (Ser 1177), phosphorylated eNOS (Thr495) and NOS2 (bottom) are shown. In all graphs, protein content was normalized with β-actin. *Arg1^fl/fl^/Tie2-Cre^tg/−^* mice are described as Arg1^-/-^ in the legend above the lanes.

Basal NO concentration in the jejunum was comparable in control mice and mice with a deletion in hematopoietic cells of arginase-1 (control versus *Arg1^fl/fl^/Tie2-Cre^tg/−^* P = 0.11, n = 5; **Figure 3A**). NO concentration was significantly lower during LPS infusion in jejunal tissue of control mice (Control + LPS). In contrast, a ∼3.5-fold higher jejunal NO concentration was seen in LPS-treated *Arg1^fl/fl^/Tie2-Cre^tg/−^ mice* (P<0.001, n = 5; **Figure 3A**). In line, the NO production measured with two-photon fluorescence microscopy in endothelial cells of LPS-treated *Arg1^fl/fl^/Tie2-Cre^tg/−^* mice, exhibited a significant higher *ex vivo* NO concentration than endothelial cells of LPS-treated control mice (**Figure S3 in File S1**). To identify the specific NOS protein involved, we measured the protein concentration of NOS2 and NOS3 in jejunal tissue and the degree of phosphorylation of NOS3 on Ser1177, a parameter for NOS3 activation, and on Thr495, a parameter for NOS3 inhibition. While total tissue concentrations of NOS3 did not differ between groups (**Figure 3B**), LPS treatment decreased NOS3 Ser1177 phosphorylation in control mice and tended to be higher in *Arg1^fl/fl^/Tie2-Cre^tg/−^* mice (**Figure 3C**). Under basal conditions, Thr495 phosphorylation was significantly lower in *Arg1^fl/fl^/Tie2-Cre^tg/−^* mice than control mice (**Figure 3D**). Thr495 phosphorylation did not differ significantly between groups treated with LPS infusion. The last finding suggests that NOS3 activity was not inhibited during LPS infusion in *Arg1^fl/fl^/Tie2-Cre^tg/−^* mice (**Figure 3D**). However, *Nos3* mRNA expression did not follow this pattern, as *Nos3* was significantly lower in *Arg1^fl/fl^/Tie2-Cre^tg/−^* mice treated with LPS compared to basal conditions (**Figure 2C**).

**Figure 4:**
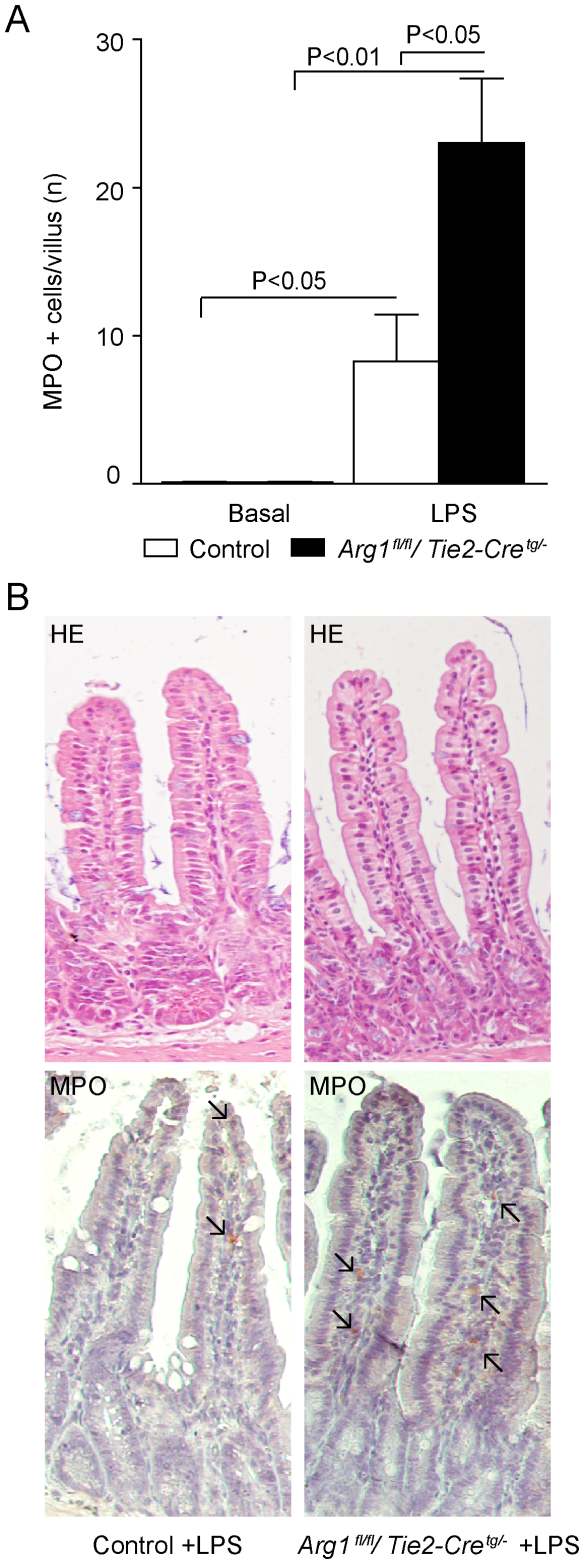
LPS treatment increases density of MPO-positive cells in *Arg1^fl/fl^/Tie2-Cre^tg/−^* mice. (A) LPS infusion increased the influx of MPO-positive cells into jejunal tissue of control and *Arg1^fl/fl^/Tie2-Cre^tg/−^* mice, but more so in *Arg1^fl/fl^/Tie2-Cre^tg/−^* mice (P<0.05). Magnification 200x. (B) Jejunal villi of H&E- (upper panel) and MPO-stained (lower panel) villi of LPS-treated control (left) and *Arg1^fl/fl^/Tie2-Cre^tg/−^* mice (right). Arrows indicate the MPO positive cells. Magnification 200x.

LPS treatment increased NOS2 protein concentration in both *Arg1^fl/fl^/Tie2-Cre^tg/−^* and control mice (p<0.05; **Figure 3E**), combined with a trend toward increased *Nos2* mRNA expression (**Figure 2D**). Furthermore, in all LPS-treated mice an enhanced influx of inflammatory cells (cells known to mediate the NOS-induced response to LPS [40]) was found, reflected by an increased number of myeloperoxidase (MPO)-positive cells per villus (**Figure 4A, B**). These results may indicate an increased inflammatory response, as suggested by an enhanced NOS2 protein concentration, an enhanced NO production and increased influx of inflammatory cells in LPS-treated *Arg1^fl/fl^/Tie2-Cre^tg/−^* mice.

### In vitro NOS2-derived NO production and inflammation in macrophages of *Arg1^fl/fl^/Tie2-Cre^tg/−^* mice

**Figure 5:**
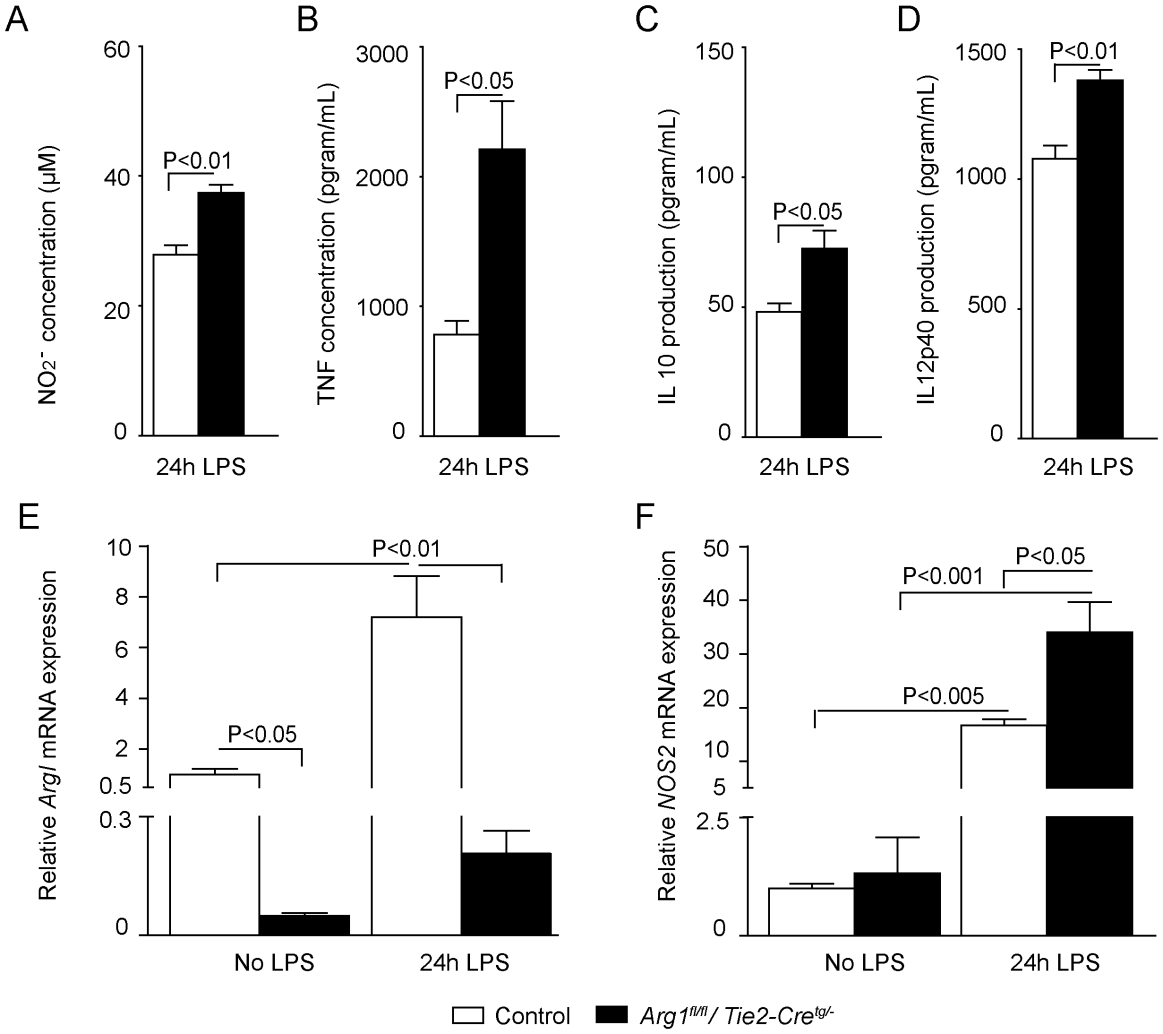
Enhanced nitrite, TNF, IL-10 and IL-12p40 production and *Nos2* mRNA abundance in cultured *Arg1^fl/fl^/Tie2-Cre^tg/−^* bone marrow-derived macrophages after LPS stimulation. LPS-treated bone marrow-derived macrophages from *Arg1^fl/fl^/Tie2-Cre^tg/−^* mice (n = 3) produce more nitrite (A; P<0.01), TNF (B: P<0.05), IL-10 (C;P<0.05) and IL-12p40 (D; P<0.01) in 24 hrs than macrophages from control mice. (E) LPS treatment increased *Arg1* mRNA abundance in control macrophages (P<0.01; n = 3). Note that the *Arg1* mRNA expression was >20-fold lower in *Arg1^fl/fl^/Tie2-Cre^tg/−^* than in control macrophages (n = 3, P<0.05), both under basal conditions and after LPS treatment. (F) Addition of LPS increased *Nos2* mRNA abundance in both *Arg1^fl/fl^/Tie2-Cre^tg/−^* and control macrophages (P<0.001 and P<0.005, respectively; n = 3, both groups), but the increase was more prominent in the *Arg1^fl/fl^/Tie2-Cre^tg/−^* macrophages (P<0.05).

To characterize the inflammatory response in *Arg1^fl/fl^/Tie2-Cre^tg/−^* animals, we obtained bone marrow-derived macrophages (BMMs) of control and *Arg1^fl/fl^/Tie2-Cre^tg/−^* mice and cultured these under basal and endotoxemic conditions. Stimulation with LPS for 24 hours resulted in significantly increased nitrite (NO_2_^−^) production in *Arg1^fl/fl^/Tie2-Cre^tg/−^* versus control BMMs (**Figure 5A**), reflecting an enhanced NOS2- derived NO production. *Arg-1* deficiency also resulted in an increased inflammatory profile with an increased production of the cytokines TNF (>2-fold), IL10 (∼1.5-fold) and IL12p40 (∼1.2-fold) in cultured *Arg1^fl/fl^/Tie2-Cre^tg/−^* versus control BMMs (**Figure 5B–D**). In addition, after LPS stimulation, *Arg1^fl/fl^/Tie2-Cre^tg/−^* BMMs showed an enhanced *Nos2* mRNA expression and as expected combined with an impaired upregulation of *Arg-1* mRNA compared to control BMMs (**Figure 5E, F**).

### Increased arginine concentration and vascular NO production do not improve jejunal microcirculation in *Arg1^fl/fl^/Tie2-Cre^tg/−^* mice

**Figure 6:**
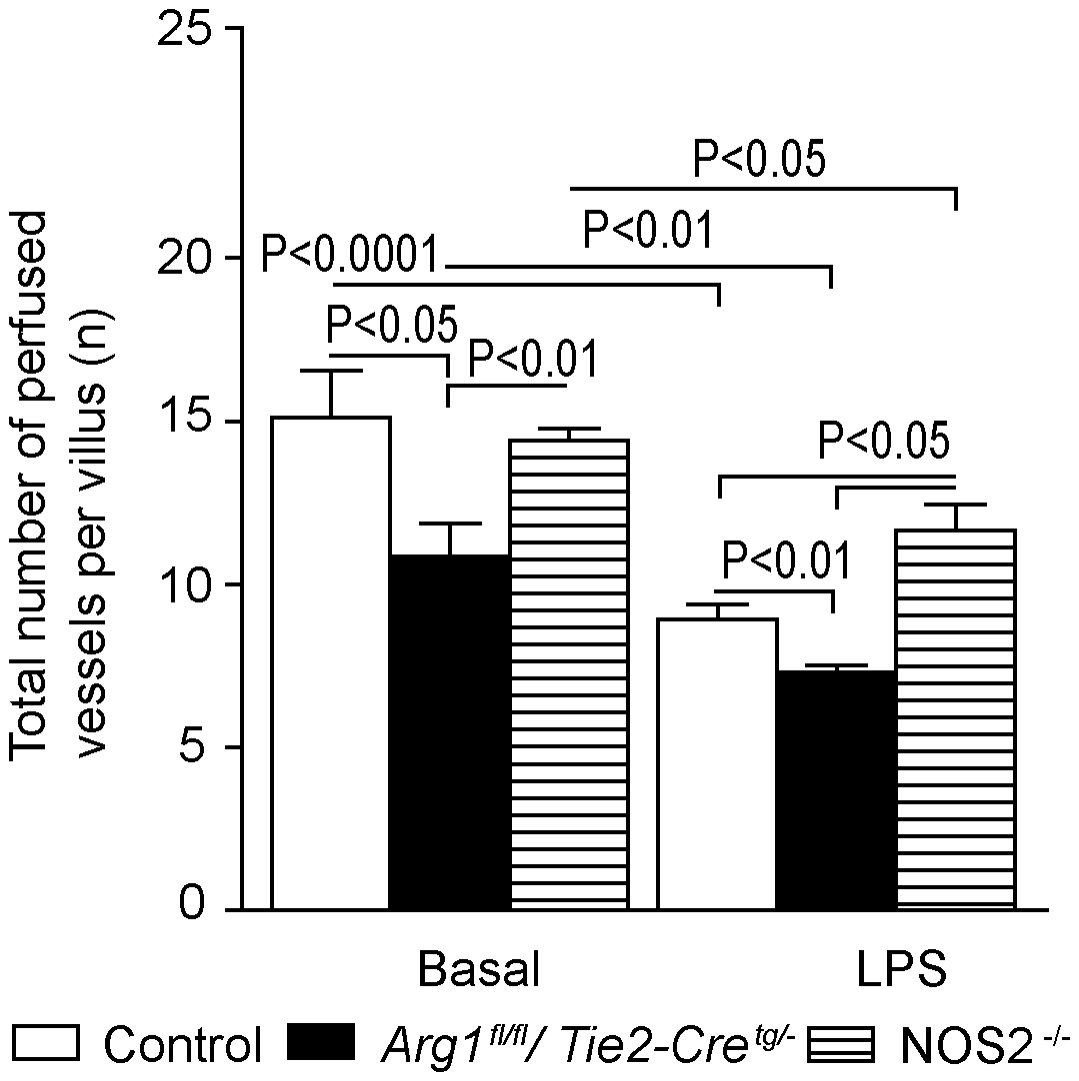
Number of perfused vessels per villus under basal conditions and after LPS treatment in control, *Arg1^fl/fl^/Tie2-Cre^tg/−^* and *Nos2^−/−^* mice. Number of perfused vessels was determined with SDF-imaging. Fewer vessels per villus were perfused in *Arg1^fl/fl^/Tie2-Cre^tg/−^* mice than in control and *Nos2^−/−^* mice, both under basal conditions and after LPS treatment. LPS infusion decreased the number of perfused vessels per villus in all three mouse strains (all n = 7).

Under basal conditions, tissue-specific *Arg-1* deficient mice had a significantly lower number of perfused vessels per villus in the jejunal microcirculation than control mice (P<0.05, n = 7, **Figure 6**). LPS infusion resulted in a significant decrease in the total number of perfused vessels per villus compared with basal conditions in control and *Arg1^fl/fl^/Tie2-Cre^tg/−^* mice (**Figure 6**). This difference between control and *Arg1^fl/fl^/Tie2-Cre^tg/−^* mice under basal conditions was also present after LPS infusion, where the total number of perfused vessels per villus was again significantly lower in *Arg1^fl/fl^/Tie2-Cre^tg/−^* than in control mice (P<0.01). Under basal conditions, the microvascular flow index (MFI), a determination of the predominant type of flow in the villi, indicated normal flow (MFI = 3) in all groups (**Supplementary Materials and Methods in File S1**). Endotoxemia resulted in a significantly decreased MFI in the LPS control group compared with basal conditions (control versus control + LPS, 3.0±0.0 versus 2.1±0.1, respectively, n = 7; P<0.0001). Endotoxemia in *Arg1^fl/fl^/Tie2-Cre^tg/−^* mice also resulted in a significantly decreased MFI when compared with basal conditions (1.5±0.2 versus 3.0±0.0, respectively, n = 7; P<0.0001). However, the MFI in the *Arg1^fl/fl^/Tie2-Cre^tg/−^* mice + LPS group was also significantly lower than that in the control + LPS group (P<0.05). Moreover, LPS infusion resulted in a significantly lower percentage of perfused villi in the *Arg1^fl/fl^/Tie2-Cre^tg/−^* + LPS group than in the control + LPS group (P<0.01) (**Figure S2 in File S1**).

### NOS2 inhibition enhances perfusion in *Arg1^fl/fl^/Tie2-Cre^tg/−^* mice

To test whether the negative effect of *Arg-1* deficiency on the microcirculation was dependent on increased NOS2-mediated NO production, we treated *Arg1^fl/fl^/Tie2-Cre^tg/−^* mice with the selective NOS2 inhibitor 1400 W [38]. *In vivo* administration of 1400 W completely eliminated extra NO production in LPS-treated *Arg1^fl/fl^/Tie2-Cre^tg/−^* mice and resulted in NO concentrations comparable to basal concentrations (*Arg1^fl/fl^/Tie2-Cre^tg/−^* versus *Arg1^fl/fl^/Tie2-Cre^tg/−^* + LPS+1400 W, 3.75±0.6 versus 4.91±0.3 pmol MNIC/mg wet tissue, P = 0.14, n = 5), which indicates that the increased NO production was indeed NOS2-dependent. In addition, *in vivo* supplementation of the *Arg1^fl/fl^/Tie2-Cre^tg/−^* + LPS group with 1400 W resulted in a significantly decreased *ex vivo* NO production in the endothelial cells of the carotid artery as demonstrated by 2-photon microscopy (**Figure S3 in File S1**). Importantly, the NO-production observed in endothelial cells of carotid arteries of *Arg1^fl/fl^/Tie2-Cre^tg/−^* mice treated with LPS + 1400 W was comparable to that found in the same cells of control + LPS mice. These data suggest that in *Arg1^fl/fl^/Tie2-Cre^tg/−^* mice treated with LPS, endothelial NO-production is largely NOS2- and only partially NOS3-derived.

**Figure 7:**
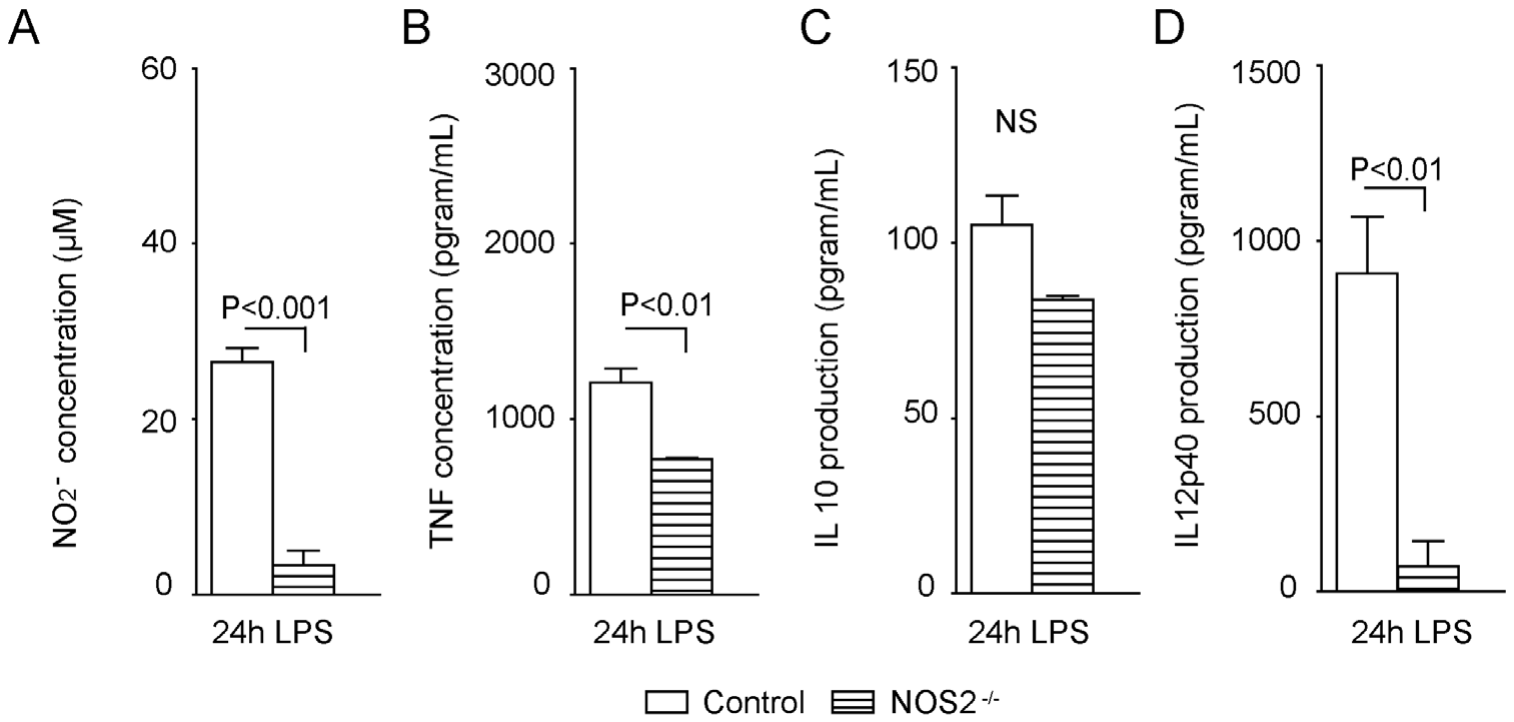
Impaired nitrite, TNF, IL-10 and IL-12p40 production in cultured *Nos2^−/−^* bone marrow-derived macrophages after LPS stimulation. (A) LPS-treated bone marrow-derived macrophages from *Nos2^−/−^* mice (n = 3) produced less nitrite (A; P<0.001), TNF (B; P<0.01), and IL-12p40 (D; P<0.01) in 24 hrs than macrophages from control mice (n = 3). IL-10 production after LPS treatment did not differ between control and *Nos2^−/−^* macrophages (C).

To further determine the role of NOS2 on the endothelial NO-production and the microcirculation during endotoxemia, *Nos2^−/−^* mice were studied. *Ex vivo* NO production measured by 2-photon microscopy in endothelial cells from these mice was comparable to that of endotoxemic control animals (*Nos2^−/−^* + LPS versus control + LPS, n = 3; P =  0.32; **Figure S3 in File S1**). As expected, BMMs from *Nos2^−/−^* mice were unable to produce substantial amounts of NO (**Figure 7A**). Furthermore, *Nos2^−/−^* BMMs showed a reduced inflammatory potential as shown by a significantly decreased production of the pro-inflammatory cytokines IL-12p40 and TNF, whereas IL-10 production was not significantly changed compared with control BMMs (**Figure 7B–D**). *In vivo*, deletion of *Nos2* improved microcirculation during endotoxemia, as shown by the higher number of perfused vessels per villus and the percentage of perfused villi in *Nos2^−/−^* mice compared to control and *Arg1^fl/fl^/Tie2-Cre^tg/−^* mice (**Figure 6** **and Figure S2 in File S1**).

## Discussion

Our study demonstrates that arginase-1 deficiency in murine endothelial cells and macrophages resulted in an increased plasma L-arginine concentration and an enhanced NO production during prolonged *in vivo* exposure to endotoxemia. Our findings reveal that the enhanced NO production in endothelial cells and jejunal tissue of *Arg1^fl/fl^/Tie2-Cre^tg/−^* mice during endotoxemia was largely NOS2-derived. This enhanced NO production was associated with an impaired intestinal microcirculation, as judged from the improved perfusion after inhibition of NOS2 activity or in *Nos2*-deficient mice exposed to endotoxemia. Accordingly, *Arg1^fl/fl^/Tie2-Cre^tg/−^* bone marrow-derived macrophages exhibited enhanced nitrite production and increased NOS2 activity, accompanied by an increased production of the inflammatory cytokines TNF and IL-12p40. Together, these findings imply an important role for arginase-1 in macrophages in the modulation of tissue inflammation and perfusion. The reduced perfusion of the intestinal mucosa of LPS-infused *Arg1^fl/fl^/Tie2-Cre^tg/−^* mice indicated that the increased circulating arginine concentration was not accessible for endothelial NOS3- mediated NO production.

Previous studies showed that arginase is a critical regulator of the availability of the substrate L-arginine for NOS-mediated NO production. This regulation plays an important role in health and under pathophysiological conditions [1], [2], [12], [22], [41], [42], such as vascular [1], [2] or inflammatory diseases [5], [6], [10], [43]. Under physiological conditions, arginase is expressed at a modest level in endothelial cells [44], [45]. Increased arginase activity, as observed in hypertension [45], ageing [2], [15], ischaemia-reperfusion [46] and inflammation [11], [12] is related to endothelial dysfunction [1], [2], [15], [28], [45]. Therefore, targeting endothelial arginase can be a promising therapeutic option to prevent vascular dysfunction caused by an impaired NOS3-mediated NO production, as present during endotoxemia. In line with previous observations [28], [47], [48], we found increased plasma arginine concentrations in *Arg1^fl/fl^/Tie2-Cre^tg/−^* mice. It is important to note that basal jejunal tissue arginine concentrations were not altered in *Arg1^fl/fl^/Tie2-Cre^tg/−^* mice when compared to control mice. Furthermore, the observed higher plasma arginine concentration in *Arg1^fl/fl^/Tie2-Cre^tg/−^* mice was not related to changes in basal NO production or jejunal microcirculation. In aggregate, all these observations indicate that, under basal conditions, NOS3-mediated NO production, does not increase due to increased plasma L-arginine. Therefore, we created an experimental L-arginine-deficient state with prolonged endotoxin infusion [31] to determine the role of the tissue-specific arginase-1 on NOS3-mediated NO during inflammation.

LPS infusion in *Arg1^fl/fl^/Tie2-Cre^tg/−^* mice did not alter plasma arginine concentrations, but resulted in significantly decreased jejunal arginine and ornithine concentrations, implying an increased tissue NO production and increased ornithine turnover. Although NO production in endothelial cells and jejunal tissue of the LPS-infused *Arg1^fl/fl^/Tie2-Cre^tg/−^* mice increased, this increased NO production did not improve the microcirculation. The enhanced arginase-2 activity in the *Arg1^fl/fl^/Tie2-Cre^tg/−^* mice after LPS infusion, may contribute to impaired microcirculation, as arginase-2 activity also constrains NOS3 activity [3], [15], [44].

Normally, arginase-1 is considered to compete with NOS2 for their mutual substrate arginine [3], [15], [49], [50] and is indirect capable of regulating NOS2 translation and expression by controlling this substrate availability, especially in the endothelium and in immune-modulating cells such as macrophages [6], [7]. As observed in our study, endothelial and macrophage arginase-1 deficiency resulted in an enhanced plasma arginine concentration during basal conditions. The decrease in NOS2 protein content in jejunal tissue of *Arg1^fl/fl^/Tie2-Cre^tg/−^* mice under basal conditions does, therefore, not correlate with the substrate availability and most likely reflects the effect of a factor not included in our analysis. Besides regulation of NOS2 at the translational level, a major part of this regulation occurs at the transcription level and mRNA stability [51]. Therefore, *Nos* mRNA abundance may differ from the protein levels based on this complex regulation of the NOS isoforms genes [9], [51]–[54]. The absence of arginase-1 during basal conditions may possibly have contributed in an altered regulation of *Nos2* at a pretranslational level, which led to the observed lower NOS2 protein content.

LPS is also capable of regulating NOS2, as LPS increases the *Nos2* mRNA abundance and protein in murine tissues [55], which was also observed in our jejunal tissue of both control and *Arg1^fl/fl^/Tie2-Cre^tg/−^* mice. Moreover, *Nos2* mRNA expression and NOS2 activity were significantly upregulated in cultured arginase-1 deficient macrophages after LPS stimulation. Furthermore, LPS treatment caused a significant higher influx of inflammatory cells into jejunal tissue of arginase-1 deficient than of control mice. Because the presence of arginase-1 in inflammatory cells restrains the inflammatory response via limitation of arginine availability for NO production [7], [56], both a reduction in NOS2 concentration and activity appear to be mechanisms to moderate inflammation. Since the remaining arginase-1 activity in *Arg1^fl/fl^/Tie2-Cre^tg/−^* mice is negligible, we further hypothesize that NOS2 rather than NOS3 benefitted from the arginase-1 deficiency. Indeed, the unrestrained NOS2 activity in endothelium and macrophages of *Arg1^fl/fl^/Tie2-Cre^tg/−^* mice most likely causes the detrimental microcirculatory outcome of LPS treatment in these animals.

*Arg1* deficiency clearly affects NOS3 activation, as phosphorylation of Ser1177 (activation mark) was enhanced and phosphorylation of Thr495 (inhibition mark) was decreased. Although total *Nos3* mRNA abundance was increased, the presence of mRNA is never a guarantee for subsequent translation into protein as there was no enhanced protein expression of NOS3 observed, demonstrating not only posttranslational but also translational control.

In the present study, cultured *Arg1^fl/fl^/Tie2-Cre^tg/−^* BMMs produced more nitrite and pro-inflammatory cytokines (TNF and IL-12p40) than control BMMs, indicating an enhanced pro-inflammatory state. In contrast, the pro-inflammatory cytokine production was impaired in *Nos2^−/−^* BMMs demonstrating the central role of NOS2 in activated macrophages. The central role of NOS2 in the inflammatory response was underscored by the protective effects of the NOS2-selective inhibitor 1400 W on tissue (endothelial cells and jejunum) NO production. These findings fit with the current paradigm of macrophage polarization in which arginase-1 characterizes the anti-inflammatory alternatively activated macrophages, whereas NOS2 is an important mediator in pro-inflammatory, classically activated macrophages [57]. We therefore, wondered whether *in vivo* NOS2 inhibition would positively influence endothelial NO production and the microcirculation. Indeed, *Nos2^−/−^* mice subjected to endotoxemia showed an improved jejunal microcirculation compared with *Arg1^fl/fl^/Tie2-Cre^tg/−^* + LPS-treated mice. This finding shows that NOS2-mediated NO production during endotoxemia compromises the microcirculation.

The main limitation of this study is that the study design does not allow differentiation between effects of arginase-1 depletion in macrophages and vascular endothelial cells *in vivo*. We, therefore, performed *in vitro* experiments to begin to understand the impact of the arginase-1 deficiency in macrophages or endothelial cells only.

In conclusion, we show that arginase-1 plays a crucial role in controlling NOS2 activity during inflammation in both endothelial cells and macrophages. Therefore, interventions with the aim to modulate arginase activity during endotoxemia *in vivo* have to be performed with great precautions. Future (mechanistic) studies need to focus on the fragile balance between arginase-1 and NOS2 under inflammatory conditions, since this balance may be the key to an improvement of vascular function.

## Supporting Information

File S1 **Detailed information about the material and methods used in this study combined with an additional result section.**
(DOC)
