# Supplementary material for: Arginase-1 Deficiency Regulates Arginine Concentrations and NOS2-Mediated NO Production during Endotoxemia
Source: PLoS One. 2014 Jan 21;9(1):e86135. doi: 10.1371/journal.pone.0086135 (PMC3897658; doi:10.1371/journal.pone.0086135)
Supplement: File S1 — Detailed information about the material and methods used in this study combined with an additional result section. (DOC) [file pone.0086135.s001.doc]

# SUPPORTING INFORMATION FILE S1

# Detrimental effects of arginase-1 deficiency on vascular function and NOS3 mediated NO production during endotoxemia

Karolina AP Wijnands, Marten A Hoeksema, Dennis M Meesters, Nynke MS van den Akker, Daniel GM Molin, Jacob J Briedé, Mitrajit Ghosh, S Eleonore Köhler, Marc AMJ. van Zandvoort, Menno PJ de Winther, Wim A Buurman, Wouter H Lamers, Martijn Poeze

**SUPPLEMENTAL MATERIAL AND METHODS**

**Amino-acid and polyamine analysis**

After blood sampling, blood (600-800μL) was collected in pre-chilled, heparinized microvettes® (Sarstedt, Nϋmbrecht, Germany) on ice, and centrifuged immediately (4°C for 15 minutes at 8,500 g). Acetonitrile was added for deproteinization, samples were vortexed and stored at -80°C and used for later amino-acid determination. Frozen homogenized tissue samples (approximately 30mg) were added to 250µL of 5% sulfosalicylic acid and 0.1g glass beads (1.0mm diameter) for deproteinization, beaten for 30 seconds with the mini-beadbeater (Biospec products) and used to determine tissue amino acid and polyamine concentrations. Amino-acid concentrations in plasma and tissue, and tissue polyamine concentrations were determined by a fully automated liquid chromatography-mass spectrometry system (LC-MS, Thermoquest LTQ, Veenendaal, the Netherlands) as described before[1].

*In vivo tissue NO measurements*

The *in vivo* NO concentrations in jejunal tissue (n=25) were quantified by NO-trapping with Fe^2+^-dithiocarbamate complexes as mono-nitrosyl iron complexes (MNIC) and measured with electron spin resonance (ESR) spectroscopy as described elsewhere [2,3]. NO concentrations were calculated from the height of the three-line NO amplitude with Bruker WINEPR software as described previously [2].

*Jejunal microcirculation measurements with SDF imaging*

The mucosal microcirculation in the jejunal villi was microscopically visualized with the side-stream dark field (SDF) imager (Microscan, Amsterdam, the Netherlands) [4,5], which has been described in detail before [2]. In brief, mice (n=42) were anaesthetized, their peritoneal cavities opened and a small part of the jejunum exteriorized. A small longitudinal incision (0.5 cm) in the jejunal segment was made to microscopically visualize the mucosa with a sidestream dark-field (SDF) imager (Microscan, Amsterdam, the Netherlands) [4,5]. A specially designed stand was used to stabilize the SDF imager and to prevent pressure on the tissue or camera movement during the measurements. Circulation, determined as the total number of perfused vessels per villus was analyzed using Automated Vascular Analysis software 3.0 (Microscan, Amsterdam, the Netherlands), adjusted according to de Backer et al. [6-8]. Furthermore, the average microvascular flow index (MFI), the determination of the predominant type of flow in the villi in the four quadrants of the image was determined (described as 0 = absent, 1 = intermittent, with at least 50% of the time no flow, 2 = sludging, 3 = normal or 4 = hyperdynamic flow) [7]. All imaging experiments were done by an experienced investigator. Images were analyzed by 2 independent experienced researchers.

**Bone marrow-derived macrophage culture experiments**

To determine the response of *Arg-1*-deficient macrophages under basal and endotoxemic conditions, bone marrow cells of control (n=6), *Arg1^fl/fl^/Tie2-Cre^tg/-^* mice (n=3) and *NOS2^-/-^* deficient mice (n=3), were isolated from femurs and tibiae of the mice to obtain bone marrow-derived macrophages (BMM). Cells were cultured in 24-well plates (Greiner) with RPMI-1640 supplemented with 10% heat-inactivated fetal calf serum, penicillin (100 U/ml), streptomycin (100 μg/ml), and L-glutamine 2mM (all Gibco, Life Technologies), with the addition of 15% L929-cell–conditioned medium for 8 days to generate bone marrow-derived macrophages (BMMs), as described previously [9]. To determine the response to inflammation, BMMs of control (3 *Arg1^fl/fl^* and 3 *NOS2^+/+^* mice, littermates of the respective knockout strains), *Arg1^fl/fl^/Tie2-Cre^tg/-^* and *NOS2^-/-^* mice were activated with 10 ng/mL LPS (Sigma) for 24 hours. Cell homogenates were used to measure arginase activity [10]. Nitrite (NO^2-^) production was determined in cell culture supernatants with Griess reagent. Concentrations of the cytokines TNF, IL-10 and IL-12p40 were determined with ELISA, according to the manufacturer’s instructions (Invitrogen, Life Technologies).

**2-photon ex vivo NO-production measurements in carotid arteries**

Endothelial NO production during endotoxemia was measured in carotid arteries of control (n=6) and *Arg1^fl/fl^/Tie2-Cre^tg/-^* mice (n=6) treated with LPS. To specify the origin of the NO produced in *Arg1^fl/fl^/Tie2-Cre^tg/-^* endothelial cells (NOS3 or NOS2), NO production in carotid arteries was either inhibited pharmacologically (1400W; n=5) or by genetic ablation of NOS2 (n=3). The 2-photon NO measurements and the microscopic setup for imaging have been described previously [11-12]. In brief, carotid arteries were excised and mounted and pressurized in a perfusion chamber (IDEE BV, Maastricht, The Netherlands) [11]. Vessels were pre-loaded with the fluorescent copper-based NO probe for 5 minutes after which basal and arginine-stimulated fluorescent measurements were performed using a Leica ultrafast TCS SP5 multiphoton microscope integrated with DM6000 CF system, DFC360FX camera system and AOBS (Leica, Mannheim, Germany). Images were analyzed using LASAF acquisition software (Leica, Mannheim Germany).

**Tissue embedding, sectioning and staining**

After collection, jejunal tissue was fixed in 4% formaldehyde and embedded in paraffin and 4μm sections were prepared for histological and immunohistochemical staining. Haematoxylin and eosin (H&E) staining was performed for morphological quantification. Neutrophils were identified by MPO (myeloperoxidase) staining (polyclonal antibody A0398, DakoCytomation, Glostrup, Denmark). Sections were deparaffinized, rehydrated and blocked for 1 hour with 5% BSA (bovine serum albumin) in Tris-buffered saline (TBS) at room temperature. Sections were incubated for 1 hour at room temperature with the primary antibody (diluted in TBS containing 0,1% BSA). After washing, specific antibody-binding was detected using a HRP (horseradish peroxidase)-labeled goat α rabbit IgG antibody (Jackson Immunoresearch, West Grove, PA). Staining was visualized with 3-amino-9-ethylcarbazole (AEC) and followed by nuclear counter staining with haematoxylin (Sigma, St. Louis, MO).

**RNA and protein isolation**

RNA and protein in macrophage and tissue samples were isolated with the AllPrep DNA/RNA/Protein kit (Qiagen, Hilden, Germany) according to the manufacturer’s protocol. In brief, jejunal samples were crushed in liquid nitrogen with a pestle and mortar, and homogenized in lysis buffer, containing β-mercaptoethanol (Promega, Madison, WI) using the Ultra Turrax Homogenizer (IKA, Labortechnik, Staufen, Germany). RNA was bound using the RNeasy spin columns, the columns were washed and RNA eluted in RNase-free water. Proteins were precipitated in the flow-through. Protein precipitate was centrifuged, and the pellet dissolved in 5% SDS. Samples were stored at - 80°C until further analysis.

**Real-Time PCR**

qPCR was performed to analyze expression of *Nos3, Nos2, Ass, Arg1, Arg2 Actb (β-actin)* and *Ppia (cyclophilin A)* genes per individual cDNA sample. DNAse (Promega) treatment of the RNA samples was performed to ensure the removal of genomic DNA. RNA quantity per sample was determined with NanoDrop spectrophotometry (Witec AG, Heitersheim, Germany). iScript cDNA synthesis kit (Biorad Laboratories, Hercules, CA, USA) was used for total cDNA synthesis. A total volume of 20 μL volume containing 10ng cDNA, one unit of IQ SYBR Green Supermix (Bio-Rad Laboratories, Hercules, CA, USA) and 300nmol/L of gene-specific forward and reverse primers for *Actb, Ppia, Nos2, Nos3, Arg1* and *Arg2* were used to perform the qPCR reactions. The primer sequences are listed in **Supplementary** **Table S1**. A three-step program was used to amplify the cDNA with the MyiQ system (Bio-Rad), which included 40 cycles of 10s at 95 °C, 20s at 60 °C and 20s at 70 °C. Melting curve analysis was performed to determine the specificity of the amplification. To determine the gene expression levels, iQ5 software was used with a ∆Ct relative quantification model. The relative expression of the two household genes *β-actin* and *cyclophilin A* was used to calculate the geometric mean and served as a normalization factor.

**Western blot analysis**

Sample protein concentrations were determined using the BCA protein assay kit (Pierce, Etten-Leur, the Netherlands). Ten µg of total protein per sample were heated in SDS sample buffer containing β-mercaptoethanol to ensure complete denaturation and loaded on a 10% polyacrylamide gel. Nitrocellulose membranes (Bio-Rad Laboratories, Hercules, CA, USA) were used to blot the different gels overnight. Membranes were blocked for 1 hour in 3% milk solution containing 0.05% Tween20 and incubated for 1 hour with rabbit polyclonal anti-mouse NOS2 (Abcam, Cambridge, MA, USA), rabbit polyclonal anti-mouse phosphorylated NOS3 Ser 1177 (Cell signaling technology, Danvers, MA) and rabbit polyclonal anti-mouse phosphorylated NOS3 Thr495 (Santa Cruz, biotechnology, Dallas, Texas, USA), respectively, at room temperature. After washing, membranes were incubated with the proper corresponding secondary antibody, goat anti-rabbit HRP-conjugated antibody for NOS2, NOS3 and phosphorylated NOS3 (Jackson). The membranes were re-probed with anti-mouse β-actin (Sigma) and rat anti-mouse HRP-conjugated secundary antibody (Jackson) to confirm equal loading and transfer of the samples. A chemiluminescence reaction with a home-made chemiluminescence solution containing luminol, p-coumaric acid and H_2_O_2_ was used to capture the signals on X-ray film (Fuji SuperRX, Tokyo, Japan). The expression of the household gene *β-actin* was used as a normalization factor.

**SUPPLEMENTAL RESULTS**

*Intracellular polyamine concentrations*

Since ornithine is stated to be an important precursor of polyamine synthesis [13], we measured the intracellular polyamine concentrations of putrescine, spermidine and spermine under basal and inflammatory conditions in control and the endothelial and macrophage specific *Arg1^fl/fl^/Tie2-Cre^tg/-^* mice **(Figure S1).** Intracellular putrescine was significantly decreased in *Arg1^fl/fl^/Tie2-Cre^tg/-^* versus control mice under basal conditions (*Arg1^fl/fl^/Tie2-Cre^tg/-^* versus control, 90.3±16.7 versus 234.3±33.3 nmol/mg wet jejunal tissue, P<0.01, n=7) (**Figure S1A**). Prolonged endotoxemia resulted in a significantly increased intracellular putrescine concentration versus basal conditions (*Arg1^fl/fl^/Tie2-Cre^tg/-^* + LPS versus *Arg1^fl/fl^/Tie2-Cre^tg/-^*, 178.2±40.9 versus 90.3±16.7 nmol/mg wet tissue, P<0.05, n=7), which was comparable to the concentrations seen in the control + LPS (169.5±19.9 nmol/mg wet tissue). Spermidine concentrations were not different between control and *Arg^fl/fl^/Tie2-Cre^tg/-^* mice under basal or endotoxemic conditions (**Figure S1B**). The intracellular spermine concentration was significantly higher in the *Arg1^fl/fl^/Tie2-Cre^tg/-^* mice versus control mice (*Arg1^fl/fl^/Tie2-Cre^tg/-^* versus control, 2264±507.1 versus 1425±124.7 nmol/mg wet tissue, P<0.05, n=7; **Figure S1C**). LPS infusion abolished the increase under basal conditions lowering spermine concentrations in the knockout animals even further than in control + LPS (*Arg1^fl/fl^/Tie2-Cre^tg/-^* + LPS versus WT + LPS, 1092±188.5 versus 1437±64.11 nmol/mg wet tissue, P=0.07, n=7). This suggests an enhanced conversion of spermine into putrescine during prolonged endotoxemia in the knockout animals.

**Supplemental Table S1: Primers for genotyping and quantitative PCR**

| **Gene** | **Primer name** | **Primer sequence (5’-3’)** |
| --- | --- | --- |
| ***Arg1*** | Arg1-F | GGAGAGCCTTCCTGCACTTT |
|  | Arg1-R | GTGCCTTGGTCTACATTGAACATAC |
| ***Excised Arg1*** | Arg1-F2 | TCTAGAACTAGTGGATCACCTCAG |
|  | Arg1-R | GTGCCTTGGTCTACATTGAACATAC |
| ***Tie-2-Cre*** | Tie2-F | CGCATAACCAGTGAAACAGCATTGC |
|  | Tie2-R | CCCTGTGCTCAGACAGAAATGAGA |
| ***Arg2*** | Arg2-F | CCAGCTGCCATTCGAGAAG |
|  | Arg2-R | ATCATCTTGTGGGACATTAGTAAACTC |
| ***Nos2*** | NOS2-F | TTGCAAGCTGATGGTCAAGATC |
|  | NOS2-R | CAACCCGAGCTCCTGGAA |
| ***Nos3*** | NOS3-F | TTAATGTGGCCGTGTTGCA |
|  | NOS3-R | CTCTTGATGGAAGACAGGAGTTAGG |
| ***Actb*** | B-actin-F | GACAGGATGCAGAAGGAGATTACTG |
|  | B-actin-R | CCACCGATCCACACAGAGTACTT |
| ***Ppia*** | CycloA-F | TTCCTCCTTTCACAGAATTATTCCA |
| ***(*Cyclophilin A)** | CycloA-R | CCGCCAGTGCCATTATGG |

Abbreviations: F, forward primer; R, reversed primer.

**SUPPLEMENTARY FIGURES**

**
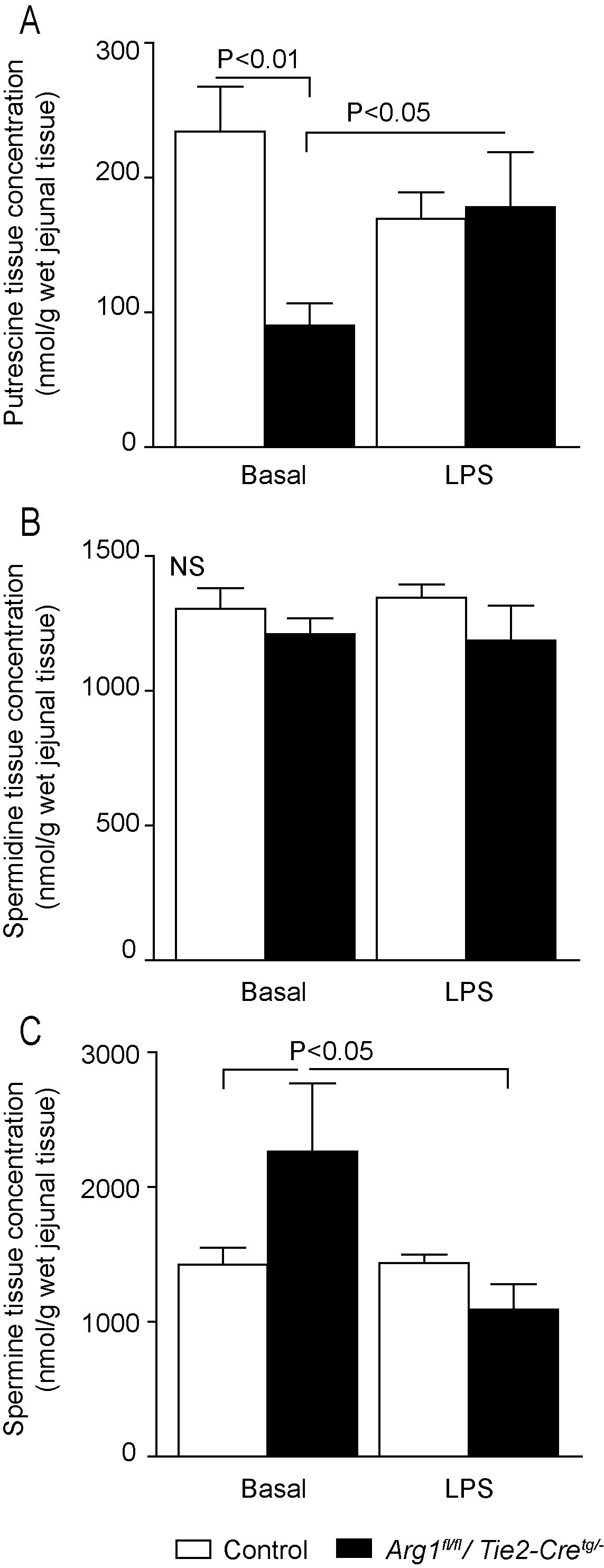
**

**Supplementary Figure S1**: **Polyamine tissue concentrations in control and *Arg1^fl/fl^/Tie2-Cre^tg/-^*****mice under physiological conditions and endotoxin infusion.** (A) Jejunal tissue putrescine concentration (nmol/g wet jejunal tissue) was significantly reduced in *Arg1^fl/fl^/Tie2-Cre^tg/-^* compared to control mice (p<0.01). LPS infusion significantly enhanced the putrescine concentration in *Arg1^fl/fl^/Tie2-Cre^tg/-^* +LPS compared to basal conditions (p<0.05). (B) Spermidine concentrations did not differ between control and *Arg1^fl/fl^/Tie2-Cre^tg/-^* mice under basal condition or endotoxin infusion. (C) Jejunal spermine tissue concentration was significantly higher in the *Arg1^fl/fl^/Tie2-Cre^tg/-^* group under basal conditions compared to the control and the *Arg1^fl/fl^/Tie2-Cre^tg/-^* + LPS group (p<0.05).


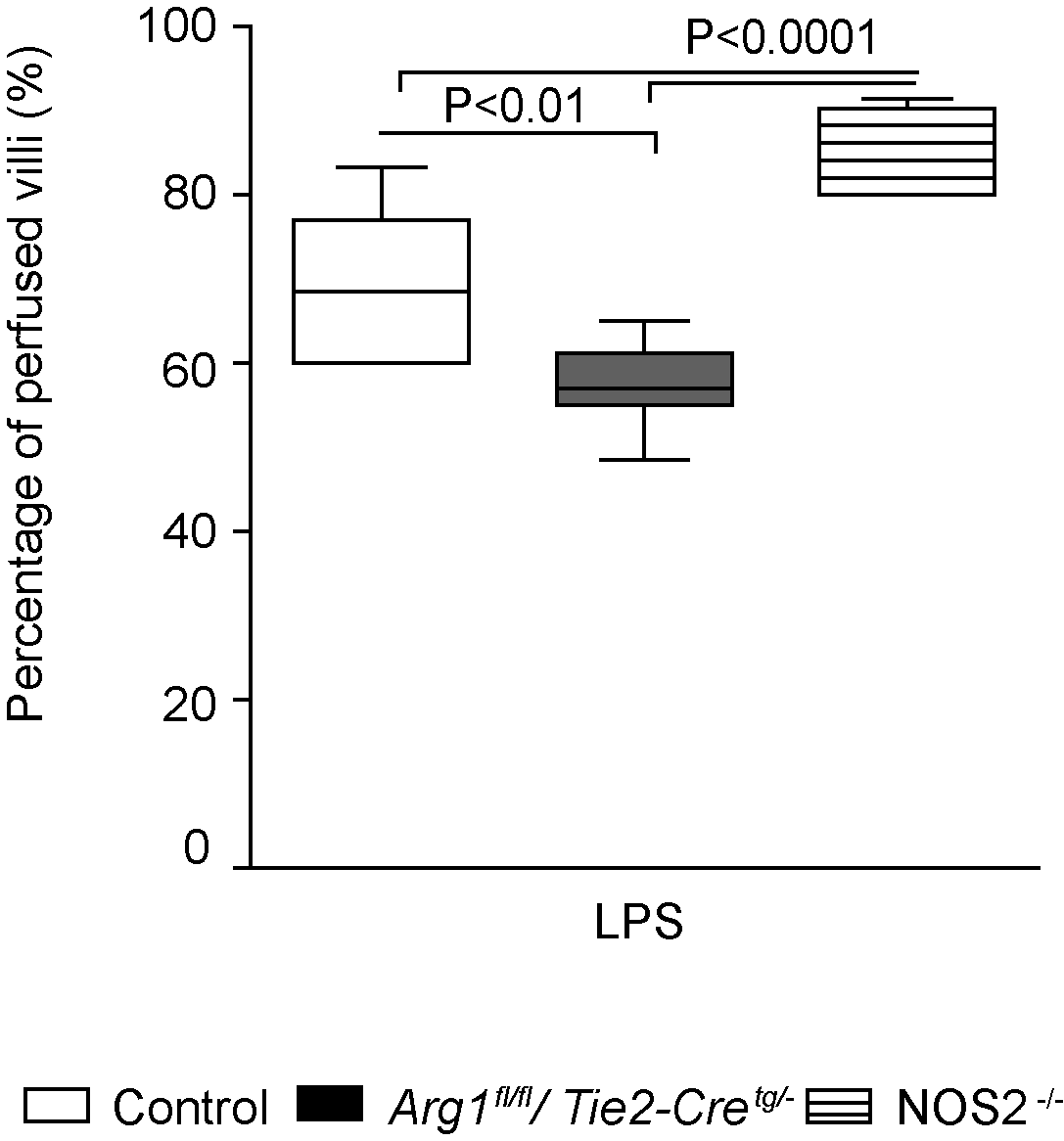


**Supplementary Figure S2**: **Circulation measurements with SDF-imaging under physiological and inflammatory conditions in control, *Arg1^fl/fl^/Tie2-Cre^tg/-^* and *Nos2^-/-^* mice.** The percentage of perfused villi was significantly reduced in the *Arg1^fl/fl^/Tie2-Cre^tg/-^* + LPS group compared to endotoxemic control and *Nos2^-/-^* groups (all n=7). Data were normalized for the number of perfused vessels during basal conditions per group.

**
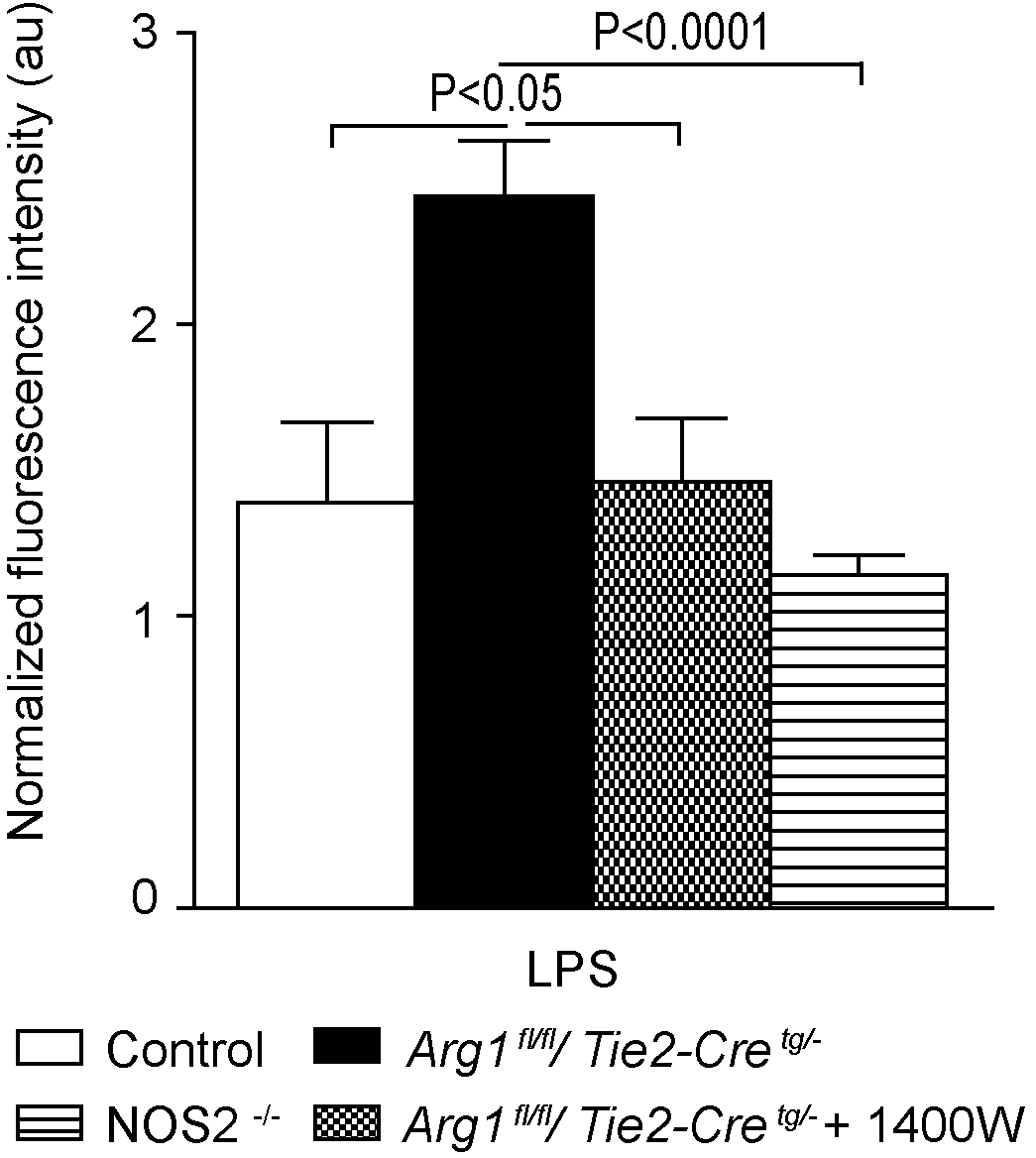
**

**Supplementary Figure S3**: **Ex vivo NO production measured in carotid arteries of control and *Arg1^fl/fl^/Tie2-Cre^tg/-^*mice.** Endothelial cells in carotid arteries of *Arg1^fl/fl^/Tie2-Cre^tg/-^* mice exhibited significantly more *ex vivo* NO production than endothelial cells of control and *Nos2^-/-^* mice during LPS treatment. *In vivo* treatment with 1400W and LPS of *Arg1^fl/fl^/Tie2-Cre^tg/-^* mice resulted in less *ex vivo* NO production compared to the *Arg1^fl/fl^/Tie2-Cre^tg/-^* + LPS group. NO production displayed as normalized fluorescence intensity (AU) and fold increase compared to basal NO production.

**SUPPLEMENTARY REFERENCES**

1. van Eijk HM, Rooyakkers DR, Deutz NE (1993) Rapid routine determination of amino acids in plasma by high-performance liquid chromatography with a 2-3 microns Spherisorb ODS II column. J Chromatogr 620: 143-148.

2. Wijnands KAP, Vink H, Briedé JJ, van Faassen EE, Lamers WH, et al. (2012) Citrulline a More Suitable Substrate than Arginine to Restore NO Production and the Microcirculation during Endotoxemia PLoS One 7: e37439.

3. van Faassen EE, Koeners MP, Joles JA, Vanin AF (2008) Detection of basal NO production in rat tissues using iron-dithiocarbamate complexes. Nitric Oxide 18: 279-286.

4. Groner W, Winkelman JW, Harris AG, Ince C, Bouma GJ, et al. (1999) Orthogonal polarization spectral imaging: a new method for study of the microcirculation. Nat Med 5: 1209-1212.

5. Spronk PE, Ince C, Gardien MJ, Mathura KR, Oudemans-van Straaten HM, et al. (2002) Nitroglycerin in septic shock after intravascular volume resuscitation. Lancet 360: 1395-1396.

6. De Backer D, Hollenberg S, Boerma C, Goedhart P, Buchele G, et al. (2007) How to evaluate the microcirculation: report of a round table conference. Crit Care 11: R101.

7. Boerma EC, Mathura KR, van der Voort PH, Spronk PE, Ince C (2005) Quantifying bedside-derived imaging of microcirculatory abnormalities in septic patients: a prospective validation study. Crit Care 9: R601-606.

8. Verdant CL, De Backer D, Bruhn A, Clausi CM, Su F, et al. (2009) Evaluation of sublingual and gut mucosal microcirculation in sepsis: a quantitative analysis. Crit Care Med 37: 2875-2881.

9. Goossens P, Gijbels MJ, Zernecke A, Eijgelaar W, Vergouwe MN, et al. (2010) Myeloid type I interferon signaling promotes atherosclerosis by stimulating macrophage recruitment to lesions. Cell Metab 12: 142-153.

10. Chang CI, Liao JC, Kuo L (1998) Arginase modulates nitric oxide production in activated macrophages. Am J Physiol 274: H342-348.

11. Megens RT, Reitsma S, Schiffers PH, Hilgers RH, De Mey JG, et al. (2007) Two-photon microscopy of vital murine elastic and muscular arteries. Combined structural and functional imaging with subcellular resolution. J Vasc Res 44: 87-98.

12. McQuade LE, Ma J, Lowe G, Ghatpande A, Gelperin A, et al. (2010) Visualization of nitric oxide production in the mouse main olfactory bulb by a cell-trappable copper(II) fluorescent probe. Proc Natl Acad Sci U S A 107: 8525-8530.

13. Deignan JL, Livesay JC, Shantz LM, Pegg AE, O'Brien WE, et al. (2007) Polyamine homeostasis in arginase knockout mice. Am J Physiol Cell Physiol 293: C1296-1301.
